# The image of a healthcare specialist on social media during the COVID-19 pandemic as a factor in the psychological stability of Internet users

**DOI:** 10.1192/j.eurpsy.2022.963

**Published:** 2022-09-01

**Authors:** L. Shaigerova, Y. Zinchenko, O. Vakhantseva

**Affiliations:** Lomonosov Moscow State University, Psychology, Moscow, Russian Federation

**Keywords:** Covid-19, healthcare specialist, social media, psychological stability of Internet users

## Abstract

**Introduction:**

As publications on social media have become an important regulator of group opinions, moods and psychological well-being during the spread of COVID-19, it seems necessary to study the contribution of various information as a source of preserving the psychological resources of the population.

**Objectives:**

To analyze the content in communities created during the spread of the COVID-19 pandemic in order to assess the image of health professionals on social media.

**Methods:**

Using datamining methods, we analyzed publications about medical staff in a large community ‘StopCoronavirus.RF’ which unites more than 400 thousand users.

**Results:**

More than 700 thousand entries were ‘liked’ in the community from March 2020 to March 2021; more than 125000 thousand posts were shared, about 290 thousand comments were written, and all community publications were viewed more than 3 billion times. Publications about medical staff working in the ‘red zones’ in Russia are distinguished by the largest number of ‘likes’ (more than 5000) and comments (more than 300). In the publications and comments, the model of modern heroes saving human lives is emphasized. This type of information contributes to the reduction of anxiety and promotion of psychological safety in case of COVID-19 contraction through establishing confidence in the competence of medical staff and their involvement in the fight against the pandemic.

**Conclusions:**

These results suggest that the image of a doctor on social media during the pandemic has acquired special value for the population. Publications on the work of medical staff in the ‘red zones’ help to cope with anxiety associated with the COVID-19 pandemic. The study was funded by RFBR, project number 20-04-60174.

**Disclosure:**

No significant relationships.

